# Psychological Assessment and Psychosocial Outcomes in Bariatric Surgery Candidates: A Retrospective Study

**DOI:** 10.3390/healthcare13111294

**Published:** 2025-05-29

**Authors:** Maria Rosaria Magurano, Daniele Napolitano, Mattia Bozzetti, Alessio Lo Cascio, Lorenzo Oppo, Laura Antonella Fernandez Tayupanta, Serena Ferrazzoli, Lucia Lopasso, Emanuela Rellini, Marco Raffaelli, Daniela Pia Rosaria Chieffo

**Affiliations:** 1 Clinical Psychology Unit, Fondazione Policlinico Gemelli IRCCS, 00168 Rome, Italy; mariarosaria.magurano@policlinicogemelli.it (M.R.M.); emanuela.rellini@guest.policlinicogemelli.it (E.R.); danielapiarosaria.chieffo@policlinicogemelli.it (D.P.R.C.); 2 CEMAD IBD UNIT, Fondazione Policlinico Universitario Agostino Gemelli IRCCS, 00168 Rome, Italy; 3 Direction of Health Professions, ASST Cremona, 26100 Cremona, Italy; mattia.bozzetti@asst-cremona.it; 4 Direction of Health Professions, La Maddalena Cancer Center, 90146 Palermo, Italy; 5 Department of Life Sciences and Public Health, Università Cattolica del Sacro Cuore, 00168 Rome, Italy; lorenzo219@hotmail.it (L.O.); la.antonellafernandez@gmail.com (L.A.F.T.); ferrazzoliserena@gmail.com (S.F.); lucia.lopasso@gmail.com (L.L.); 6 National Centre of Service and Research for the Prevention of Blindness and Vision Rehabilitation of the Visually Impaired-IAPB ETS, Fondazione Policlinico Universitario A. Gemelli, IRCCS, 00168 Rome, Italy; 7 Division of Metabolic and Endocrine Surgery, Fondazione Policlinico Universitario Agostino Gemelli, IRCCS, 00168 Rome, Italy; marco.raffaelli@unicatt.it; 8 Centro di Ricerca in Chirurgia delle Ghiandole Endocrine e dell’Obesità (C.R.E.O.), Università Cattolica del Sacro Cuore, 00168 Rome, Italy

**Keywords:** bariatric surgery, obesity, psychological assessment and psychosocial outcomes

## Abstract

**Background/Objectives:** Psychological vulnerability in individuals with obesity represents a significant concern in the context of bariatric surgery. This study aimed to assess psychosocial functioning and identify the psychological, clinical, and sociodemographic predictors of impairment among patients undergoing preoperative evaluation. **Methods**: A retrospective observational study was conducted on patients referred for bariatric surgery at a single academic medical center. Data were collected through clinical interviews and validated psychometric tools: the Clinical Impairment Assessment (CIA), the Patient Health Questionnaire-9 (PHQ-9), and the Generalized Anxiety Disorder-7 (GAD-7). Robust multiple regression analysis determined associations between CIA scores and psychological and demographic factors. **Results**: A total of 688 patients were evaluated (median age: 46 years; 70.3% female). Most had a high school education (56.9%) and were employed (69%). Elevated scores on the Clinical Impairment Assessment (CIA) were significantly associated with female gender (β = 1.075, *p* = 0.029), moderate anxiety (GAD-7 ≥ 10; β = 3.85, *p* < 0.001), and severe depressive symptoms (PHQ-9 ≥ 15; β = 16.67, *p* < 0.001). Other significant predictors included prior psychotherapy (β = 1.18, *p* = 0.044), aesthetic motivation for surgery (β = 0.92, *p* = 0.120), and expectations that weight loss would improve self-esteem (β = 2.11, *p* = 0.001) or social relationships (β = 1.98, *p* = 0.002). Conversely, physical activity was associated with lower CIA scores (β = –1.23, *p* = 0.050). The regression model showed strong explanatory power (McFadden R^2^ = 0.529). **Conclusions**: This study highlights key predictors of psychosocial distress in bariatric candidates, underscoring the importance of comprehensive psychological assessment before surgery. The CIA appears to be a valuable screening and monitoring tool. Future research should explore the longitudinal evolution of psychosocial functioning and support the integration of psychological care into multidisciplinary bariatric programs.

## 1. Introduction

Obesity is a major public health concern, with its prevalence steadily increasing worldwide [1,2]. Severe obesity, defined as a body mass index (BMI) ≥ 40 kg/m^2^ or ≥35 kg/m^2^ with comorbidities, presents a significant risk for various medical conditions, including cardiovascular diseases, type 2 diabetes mellitus, and obstructive sleep apnea [1,3]. While lifestyle modifications and pharmacotherapy are considered primary interventions, bariatric surgery has emerged as the most effective treatment for long-term weight reduction and improvement of obesity-related comorbidities [4,5]. However, weight loss alone does not necessarily translate into improved psychosocial well-being, underscoring the importance of psychological assessments before and after surgery [1,6]. The psychosocial burden of obesity is well documented, with individuals often experiencing depression, anxiety, low self-esteem, and reduced quality of life [1,7,8,9]. Studies have shown that while bariatric surgery can lead to significant psychological improvements, a subset of patients continues to struggle with mood disorders, disordered eating patterns, and impaired social functioning [1,4,10]. Given these considerations, a thorough preoperative psychological assessment is crucial for identifying patients at risk of poor psychosocial outcomes post-surgery and tailoring postoperative support accordingly [11].

Preoperative psychological screening plays a crucial role in identifying individuals at risk of psychiatric complications post-surgery [12,13]. Mental health professionals are integral to bariatric programs, ensuring patients are psychologically prepared for the significant lifestyle changes accompanying surgery [14,15]. Several studies indicate that patients with preexisting mental health conditions, such as depression or anxiety, may experience either significant improvements or exacerbations following surgery [10]. Thus, continuous monitoring and psychological support are essential for optimal postoperative outcomes [16].

In addition to psychological distress, weight-related stigma and social discrimination contribute to the overall burden of severe obesity [17]. Patients undergoing bariatric surgery often report experiences of social isolation, reduced employment opportunities, and internalized weight bias, which can persist even after significant weight loss [18]. Addressing these psychosocial challenges through targeted interventions, such as cognitive-behavioral therapy and social support programs, is crucial in ensuring sustainable improvements in quality of life [14]. Therefore, understanding the factors contributing to psychosocial impairment in bariatric candidates is critical for optimizing pre- and postoperative care.

The Clinical Impairment Assessment (CIA) was selected as the primary outcome due to its strong psychometric properties and capacity to capture psychosocial impairment in domains highly relevant to bariatric candidates, including self-esteem, social functioning, and emotional regulation. While originally developed for eating disorders, the CIA has been successfully applied in studies involving patients with obesity and weight-related psychosocial distress [19,20].

This study examines the perceived distress of patients undergoing preoperative evaluation for bariatric surgery. It identifies the socio-demographic, clinical, and psychological predictors influencing this population’s perceived social distress.

## 2. Materials and Methods

### 2.1. Design

A retrospective, single-center observational study was conducted at Fondazione Policlinico Universitario A. Gemelli in Rome. The study analyzed data from obese patients who underwent psychological assessment between February 2022 and March 2023 as part of the multidisciplinary preoperative pathway for candidates for bariatric surgery.

### 2.2. Participants

A consecutive sample of patients was enrolled. After providing informed consent, participants completed an anamnestic questionnaire and a set of self-report psychometric tools following the Società Italiana di Chirurgia dell’Obesità e delle malattie metaboliche (S.I.C.OB.) national guidelines and the International Federation for the Surgery of Obesity (I.F.S.O.) international guidelines. Subsequently, a clinical psychologist conducted a clinical interview.

### 2.3. Inclusion and Exclusion Criteria

The study included participants aged over 18 who were diagnosed with class II obesity (BMI 35–39.9 kg/m^2^) or class III obesity (BMI ≥ 40 kg/m^2^) and were waiting to undergo bariatric surgery. Exclusion criteria included individuals who were pregnant or breastfeeding at the time of data collection, as well as those diagnosed with neurological disorders associated with cognitive impairment that could interfere with the reliability of the psychometric assessment.

### 2.4. Instruments

All participants completed a structured questionnaire. The first section focused on socio-demographic variables, including age, sex, marital status, educational attainment, and employment status. These variables were collected to describe the sample and explore potential associations with other clinical and psychological factors. Subsequent sections of the questionnaire gathered information on various areas relevant to obesity and its treatment. These included the onset of weight-related issues, eating behaviors, and lifestyle habits such as physical activity and smoking. Psychological and psychiatric aspects were also investigated, including self-esteem, social relationships, previous or ongoing psychotherapy, and the use of psychotropic medications. Participants were asked about their previous experiences with weight loss attempts, such as dietary regimens and nutritional, pharmacological, surgical, or psychotherapeutic interventions. Additionally, they reported their motivations for seeking bariatric surgery, whether primarily related to health, aesthetics, or both. Finally, two 10-point Likert scales were used to evaluate participants’ expectations regarding weight loss (EWLR) outcomes through surgery (1 = not at all to 10 = very much).

The Clinical Impairment Assessment (CIA) is a 16-item self-report questionnaire designed to evaluate the severity of psychosocial impairment associated with eating disorders over the past 28 days [19,21,22,23]. Each item is rated on a four-point Likert scale (0 = “not at all” to 3 = “a lot”), with a total score ranging from 0 to 48. Higher scores reflect greater levels of psychosocial impairment. A global CIA score is calculated by summing responses to all items, allowing proportional scoring for missing responses if at least 12 items are completed. The instrument also explores three subdomains of impairment: personal, social, and cognitive, providing insight into specific areas affected. The CIA offers a straightforward index of psychosocial functioning and was selected for this study, given the relevance of psychosocial distress in patients with obesity undergoing preoperative evaluation. In this study, CIA scores were analyzed independently from scores on instruments assessing eating disorder symptomatology. The CIA has demonstrated strong internal consistency (Cronbach’s α > 0.90) and convergent validity in general and clinical populations [19,21]. It has also been validated in Italian samples with obesity and eating disorders, confirming its reliability and dimensional structure. In this study, the participants completed all 16 items of the CIA. No proportional scoring for missing responses was necessary. Only fully completed questionnaires were included in the analysis.

The Patient Health Questionnaire-9 (PHQ-9) is a validated tool used to screen, diagnose, and assess the severity of depression [24,25]. Each item is rated on a scale from 0 (“never”) to 3 (“almost every day”), yielding a total score ranging from 0 to 27. The questionnaire explores symptoms experienced in the past two weeks, such as anhedonia, low mood, sleep and appetite disturbances, fatigue, poor concentration, low self-esteem, and suicidal ideation. It requires approximately 5–10 min to complete in cognitively intact patients. Severity levels were classified using standard cut-off scores: 5–9 (mild), 10–14 (moderate), and 15 or higher (severe depressive symptoms), as validated in previous research [25]. The PHQ-9 is widely validated across multiple settings, including bariatric populations, with reported internal consistency typically above 0.80 [24,25]. Its factorial structure has been confirmed in both general medical and psychiatric populations.

The Generalized Anxiety Disorder Questionnaire-7 (GAD-7) is a screening questionnaire for anxiety disorders and allows a reliable measure of the severity of the problem [26]. The patient is asked how often he/she has been bothered by each of the issues listed in the last two weeks. The score is calculated by assigning 0, 1, 2, and 3 to the response categories of never, several days, more than half of the days, and every day. The total GAD-7 score for the seven items ranges from 0 to 21. The 5, 10, and 15 scores represent reference points for mild, moderate, and severe anxiety, respectively. The GAD-7 has shown excellent psychometric performance (Cronbach’s α ≈ 0.92), and its validity and sensitivity for detecting anxiety disorders have been confirmed in multiple populations, including Italian samples [26].

### 2.5. Statistical Analysis

Before conducting the regression analysis, the distribution of continuous variables was assessed for normality using the Shapiro–Wilk test, the Kolmogorov–Smirnov test, and visual inspection of residual plots, including histograms, Q-Q plots, and residual vs. fitted plots. Given deviations from normality, continuous variables were summarized using the median and interquartile range (IQR), while categorical variables were reported as absolute frequencies and percentages (n, %). Differences between the two groups were assessed with Wilcoxon rank-sum tests and Kruskal–Wallis ANOVA between three or more groups. A robust multiple linear regression analysis examined the relationship between the dependent variable and a set of demographics, psychological, and behavioral predictors. To account for potential heteroskedasticity, heteroskedasticity-robust standard errors (Huber–White sandwich estimators) were used, ensuring that standard errors remained valid even in the presence of non-constant variance in the residuals. The model was estimated using the ordinary least squares (OLS) method with robust standard errors. The statistical significance of each predictor was assessed using *t*-tests, and *p*-values were computed accordingly. Variables with *p*-values below 0.05 were considered statistically significant. Model selection was performed using a backward stepwise procedure, optimizing the Bayesian Information Criterion (BIC). This approach iteratively removed non-significant predictors to best balance model fit and complexity. Since conventional R2 values are not directly applicable in robust regressions, model fit was assessed using the McFadden R2 derived from the squared correlation between the observed and predicted values. Additionally, an alternative measure of model fit was obtained by running an equivalent OLS regression without robust standard errors and reporting the conventional R-squared and adjusted R-squared values for reference. To assess the fit of the robust regression model, we performed a likelihood ratio test (LRT), which compared two nested models. The final model included 17 variables selected using a backward stepwise procedure based on the Bayesian Information Criterion (BIC). This approach optimized the balance between model complexity and fit. Missing data for the PHQ-9 and GAD-7 instruments were handled using pairwise deletion. Participants with incomplete CIA responses were excluded from the final analysis to ensure data integrity.

A qualified clinical biostatistician reviewed and approved all statistical procedures and interpretations to ensure methodological accuracy and robustness.

## 3. Results

### 3.1. Descriptive Analysis

The study sample consisted of n = 688 participants, with a median age of 46 years (IQR: 18). The majority were female (70.27%), and most were either married or cohabiting (57.67%). Regarding education, 56.87% of participants had completed high school, and 68.98% were employed. The median household size was three members (IQR: 2), and the median BMI of the sample was 41 (IQR: 8).

Table 1 and Table 2 provide a detailed overview of the socio-demographic and clinical characteristics of the participants.

### 3.2. Psychological and Functional Assessment

Functional impairment, as evaluated using the CIA scale, had a median score of 10 (IQR: 13). The median of EWLR was 9 (IQR: 2). Symptoms of generalized anxiety, assessed through the GAD-7 scale, had a median score of 6 (IQR: 6), while depressive symptoms, measured via the PHQ-9 scale, also had a median score of 6 (IQR: 6). The distribution of anxiety and depression symptoms is summarized in Table 3. Regarding generalized anxiety (GAD-7 scores), 41.68% of participants exhibited no symptoms, while 34.41% had mild symptoms, and 8.08% showed severe anxiety levels. Similarly, in terms of depression (PHQ-9 scores), 30.8% were asymptomatic, while 41.9% had mild symptoms, and 7.85% displayed severe depressive symptoms.

### 3.3. Correlation

The Mann–Whitney U test indicated a significant difference in CIA scores based on gender, with females exhibiting higher impairment levels compared to males (W = 37,460, *p* < 0.0001).

Multiple comparisons revealed significant differences in CIA scores across several dichotomous variables. Participants with a history of past dietary regimens had significantly different CIA scores than those without (W = 8759.5, *p* = 0.0004). Similarly, individuals who had undergone psychotherapy (W = 37,095, *p* < 0.0001) and those currently taking psychotropic medications (W = 18,296.5, *p* < 0.0001) exhibited significant differences in CIA scores.

Expectations related to weight loss benefits were also associated with CIA scores. Specifically, those who believed that weight loss would improve social relationships (W = 31,934.5, *p* < 0.0001) or self-esteem (W = 33,214.5, *p* < 0.0001) showed significant differences in impairment levels. Conversely, expectations about weight loss improving work opportunities did not yield a significant association (W = 29,631.5, *p* = 0.243). Regarding treatment initiation motives, individuals who sought treatment for aesthetic reasons had significantly different CIA scores compared to those who did not (W = 41,604, *p* < 0.0001). However, no significant differences were found for those whose primary motivation was health-related (W = 1160.5, *p* = 0.221). Other variables, including previous weight loss treatments (W = 33,306, *p* = 0.652), family history of obesity (W = 51,962, *p* = 0.073), smoking status (W = 44,893.5, *p* = 0.695), physical activity (W = 42,369.5, *p* = 0.394), and work-related weight loss expectations, did not show statistically significant associations with CIA scores.

The Kruskal–Wallis test revealed significant differences in CIA scores based on marital status (χ^2^ = 10.4, df = 3, *p* = 0.015), education level (χ^2^ = 8.69, df = 3, *p* = 0.0337), and the age of onset of weight-related issues (χ^2^ = 12.6, df = 3, *p* = 0.00553), while no significant differences were found for employment status (χ^2^ = 0.136, df = 1, *p* = 0.713).

Post hoc pairwise Wilcoxon tests with Bonferroni correction were then performed for the significant variables. For marital status, a significant difference was found between single participants and those who were married or cohabiting (*p* = 0.025), with married/cohabiting individuals exhibiting higher CIA scores. Regarding education level, patients with a middle school education showed significantly higher CIA scores compared to those with a university degree (*p* = 0.041). Regarding the age of onset of weight-related issues, participants reporting an onset in adulthood had significantly higher CIA scores compared to those with an onset during adolescence (*p* = 0.01) and childhood (*p* = 0.033).

### 3.4. Factors Associated with CIA

The analysis revealed several significant predictors of the dependent variable. Female gender was associated with an average increase of approximately 1.08 points in CIA scores compared to males (β = 1.0753, *p* = 0.029), indicating greater psychosocial impairment. Having a history of psychotherapy was associated with a 1.18-point increase in CIA scores (β = 1.1837, *p* = 0.044), suggesting greater vulnerability to psychological impairment. Expectations about the benefits of weight loss also played a key role: patients who believed that weight loss would improve their social relationships reported a 1.98-point increase (β = 1.9762, *p* = 0.002), while those expecting improvements in self-esteem had a 2.11-point increase (β = 2.1141, *p* = 0.001).

Higher expected weight loss rate (EWLR) was modestly associated with greater impairment (β = 0.3121, *p* = 0.024).

Conversely, engagement in physical activity was associated with a 1.23-point decrease in CIA scores (β = –1.2284, *p* = 0.050), suggesting a protective effect.

Regarding mental health indicators, GAD-7 scores at all levels (mild, moderate, and severe) were significantly associated with the outcome, with the most substantial effect observed in the moderate category (β = 3.8538, *p* < 0.001). Similarly, PHQ-9 scores, which measure depressive symptoms, were highly significant across all severity levels. The strongest association was found in the severe category (β = 16.6733, *p* < 0.001), followed by moderate (β = 8.7540, *p* < 0.001) and mild depression (β = 3.7271, *p* < 0.001).

**Figure 1:**
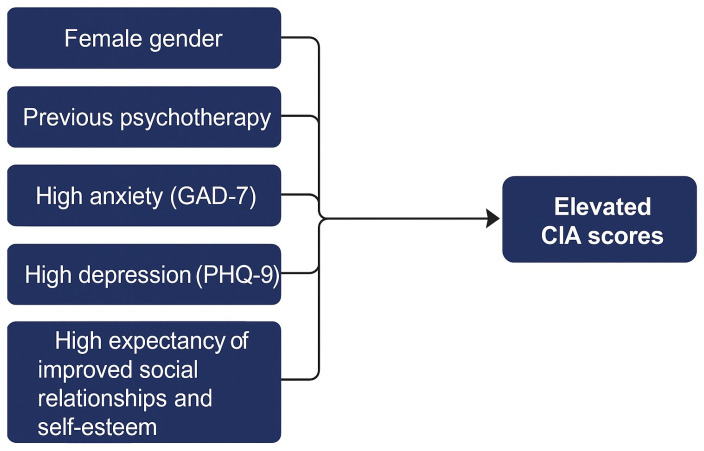
Key predictors of CIA.

The final model retained 17 predictors, selected through a backward stepwise procedure based on the BIC. The model demonstrated strong explanatory power (McFadden R^2^ = 0.529; R^2^ = 0.528; adjusted R^2^ = 0.515). The likelihood ratio test yielded χ^2^(63) = 73.784 (*p* = 0.1662), indicating that the reduced model did not significantly worsen the fit compared to the full model. The results are presented in Table 4, and a visual summary of significant predictors is shown in Figure 1.

## 4. Discussion

This study explored psychosocial functioning among individuals undergoing preoperative psychological assessment for bariatric surgery, identifying factors associated with higher levels of impairment. Framed within the biopsychosocial model of health, the study emphasizes the interaction of biological, psychological, and social domains in shaping distress in patients with obesity, a condition often characterized by hormonal imbalances, mood symptoms, and exposure to stigma and discrimination [20,26,27,28]. This comprehensive approach supports the development of multidisciplinary care pathways tailored to the complexity of this population. Evidence from endocrinology and neurobiology further supports this view, as dysregulation of the hypothalamic–pituitary–adrenal (HPA) axis and altered leptin signaling have been associated with emotional dysregulation and affective symptoms [29]. These findings suggest that future studies should consider incorporating biological profiling to better understand the physiological underpinnings of psychological vulnerability in bariatric candidates.

One of the main findings was the association between gender and psychosocial functioning [30]. Women reported significantly higher CIA scores than men, a pattern that aligns with previous literature suggesting that women with obesity experience more psychological distress due to factors such as body image dissatisfaction, internalized weight bias, and sociocultural pressure [9,31,32]. It is plausible that the higher anxiety levels observed among women in our sample, as measured by the GAD-7, may partly account for this difference. Future research could examine whether anxiety mediates the relationship between gender and psychosocial impairment.

Past psychological treatment, including psychotherapy, was also associated with greater distress. It is important to note that the “psychotherapy” variable captured both current and prior treatment experiences. This may indicate that these individuals have a longstanding vulnerability to mental health difficulties rather than experiencing acute psychological symptoms at the time of assessment [33]. This is consistent with studies documenting the chronic interplay between obesity and affective disorders [20,34]. Dieting history and motivational variables were also relevant. Individuals with a history of repeated dieting attempts exhibited higher CIA scores, possibly reflecting psychological fatigue, frustration, or diminished self-efficacy, as suggested in previous research [35,36].

In addition, participants who believed that weight loss would improve their self-esteem or social relationships reported greater levels of impairment. These expectations may reflect unmet emotional needs or overestimated psychological benefits of surgery. Such findings are consistent with studies indicating that unrealistic expectations may be associated with lower satisfaction and poorer adjustment post-surgery [1]. Addressing these beliefs during preoperative counseling may help set realistic goals and improve long-term outcomes.

Sociodemographic factors, including marital status and education, were also associated with psychosocial functioning. Specifically, married or cohabiting individuals had higher CIA scores than single participants. This association may relate to relationship strain, caregiving dynamics, or heightened sensitivity to body image in intimate contexts. Although underexplored in the literature, the influence of couple dynamics on obesity-related distress warrants further investigation [37]. Similarly, participants with lower levels of education had higher levels of impairment. This may be due to reduced health literacy, fewer psychological resources, or greater exposure to socioeconomic stressors [19]. These associations emphasize the importance of considering educational and relational contexts when evaluating psychosocial risk in bariatric populations.

We also found that individuals with adult-onset obesity reported greater psychosocial impairment compared to those with childhood or adolescent onset. This may reflect the disruptive psychological impact of weight gain during adulthood, when identity and social roles are more established [38]. Although the literature on this issue is mixed [23], adult-onset obesity may be experienced as more socially and emotionally destabilizing [22]. Surgical motivation emerged as another relevant factor. Patients who indicated aesthetic motivations for surgery had higher CIA scores than those motivated by health reasons. While both motivations are common, aesthetic-driven candidates may experience more profound dissatisfaction with body image, making them more vulnerable to distress [14,27]. These findings underscore the value of exploring motivational factors during psychological assessment. Engagement in physical activity was associated with lower levels of psychosocial impairment, consistent with literature suggesting a protective effect of exercise on mood, psychological resilience, and social functioning [14]. Promoting physical activity as part of the bariatric process may thus support both physical and mental well-being.

Notably, the strongest associations with psychosocial impairment were observed in relation to depression and anxiety symptoms, as assessed by the PHQ-9 and GAD-7. Participants with moderate or severe symptoms had markedly higher CIA scores, reinforcing the importance of screening for mental health symptoms during the preoperative process. These findings support integrating mental health professionals in multidisciplinary bariatric teams and implementing tailored psychological interventions. The CIA scores observed in our sample (median = 10, IQR = 13) are consistent with those reported in studies involving individuals with obesity [19] and significantly lower than scores typically seen in eating disorder populations [23]. This supports the scale’s sensitivity in capturing subclinical distress while confirming its applicability in bariatric contexts.

Some variables that appeared significant in the bivariate analyses, such as psychotropic medication use and aesthetic motivation, did not retain statistical significance in the multivariate model. This suggests that while these factors may be descriptively associated with psychosocial distress, their effects are attenuated when controlling for depression and anxiety symptoms, which emerge as the primary drivers of impairment.

Among the strengths of this study are the large sample size, the use of validated psychometric instruments (CIA, PHQ-9, GAD-7), and the inclusion of both clinical and sociodemographic variables, which enabled a comprehensive assessment of psychosocial functioning in a real-world bariatric population. While the cross-sectional design limits causal interpretations, the robust analytic approach—including multivariate modeling and the use of robust standard errors—enhances the internal validity of the findings. The use of self-report measures may introduce some degree of response bias; however, all instruments employed have well-established reliability and validity in clinical populations. Although the study was conducted in a single center, the large and heterogeneous sample improves its representativeness and offers valuable insights into routine clinical practice. One area of limitation concerns the lack of detailed information regarding the duration and outcomes of previous psychotherapy, which may have enriched the interpretation of psychological vulnerability. Future studies should consider prospective, multicenter designs and incorporate longitudinal follow-up to assess trajectories of psychosocial adjustment before and after surgery.

## 5. Conclusions

In conclusion, this study identifies several psychological and sociodemographic factors that contribute to elevated psychosocial impairment among candidates for bariatric surgery. Mood and anxiety symptoms, prior psychological treatment, repeated dieting attempts, unrealistic expectations, and specific sociodemographic characteristics—such as female gender, lower education, and adult-onset obesity—emerged as key risk factors. The findings underscore the need for comprehensive psychological assessment protocols that include evaluation of expectations, motivations, and psychiatric comorbidities. While the CIA instrument shows promise as a screening and monitoring tool, its use should be further evaluated in longitudinal contexts. Importantly, psychological support should not be limited to the preoperative phase but integrated throughout the multidisciplinary bariatric care continuum. Future research should adopt prospective, multicenter, and mixed-method approaches to better understand the evolution of psychosocial functioning and to develop tailored, evidence-based interventions to address the complex psychological needs of this population.

## Figures and Tables

**Table 1:** Socio-demographic variables.

Variable		Frequency	Percentage
Gender	Male	184	29.73
	Female	435	70.27
Marital status	Single	189	30.53
	Separated	67	10.82
	Married/cohabiting	357	57.67
	Widowed	6	0.97
Educational level	First school	4	0.65
	Bachelor’s degree	114	18.42
	Middle School	149	24.07
	High school	352	56.87
Employment status	No	192	31.02
	Yes	427	68.98

**Table 2:** Clinical variables.

Variable		Frequency	Percentage
Onset of weight issues	Teenaging	203	BMI (kg/m^2^) 32.79
	Adult	252	BMI (kg/m^2^) 41.11
	Infant	164	BMI (kg/m^2^) 26.49
Past dietary regimens	No	35	5.65
	Yes	584	94.35
Weight loss treatments	No	507	81.91
	Yes	112	18.09
Family history of obesity	No	258	41.68
	Yes	361	58.32
Smoking status	No	453	73.18
	Yes	166	26.82
Psychotherapy	No	377	60.90
	Yes	242	39.10
Current psychotropic drugs	No	532	85.95
	Yes	87	14.05
Weight loss expectations—social relationships	No	373	60.26
	Yes	246	39.74
Weight loss expectations—self-esteem	No	263	42.49
	Yes	356	57.51
Weight loss expectations—work	No	520	84.01
	Yes	99	15.99
Physical activity	No	476	76.90
	Yes	143	23.10
Reason for treatment initiation—health	No	1	0.16
	Yes	618	99.84
Reason for treatment initiation—aesthetics	No	233	37.64
	Yes	386	62.36

Legend: body mass index (BMI).

**Table 3:** Psychological distress.

Category	GAD-7 Score (n, %)	PHQ-9 Score (n, %)
Absent	258 (41.68%)	212 (30.8%)
Mild	213 (34.41%)	288 (41.9%)
Moderate	98 (15.83%)	132 (19.2%)
Severe	50 (8.08%)	54 (7.85%)

Legend: GAD-7: Generalized Anxiety Disorder Questionnaire-7; PHQ-9: Patient Health Questionnaire-9.

**Table 4:** Predictor of CIA.

Variable	Beta	SE	t-Value	*p*-Value
Intercept	−2.578	1.821	−1.415	0.166
Gender (female)	1.075	0.571	1.882	0.029
Past dietary regimens (yes)	2.421	1.103	2.194	0.091
Weight loss treatments (yes)	−1.085	0.666	−1.629	0.124
Psychotherapy (yes)	1.183	0.567	2.085	0.044
Current psychotropic drugs (yes)	1.245	0.786	1.592	0.156
Weight loss expectations improve social relationships (yes)	1.976	0.596	3.311	0.002
Weight loss expectations improve self-esteem (yes)	2.114	0.592	3.56	0.001
Physical activity (yes)	−1.228	0.607	−2.021	0.050
Reason for treatment initiation aesthetics (yes)	0.920	0.553	1.664	0.120
EWLR	0.312	0.153	2.037	0.024
GAD-7 (moderate anxiety)	3.853	0.910	4.233	0.000
GAD-7 (severe anxiety)	3.135	1.210	2.591	0.035
GAD-7 (mild anxiety)	1.466	0.662	2.212	0.035
PHQ-9 (mild)	3.727	0.680	5.479	<0.001
PHQ-9 (moderate)	8.754	0.925	9.461	<0.001
PHQ-9 (severe)	16.673	1.323	12.601	<0.001

Legend: beta = regression coefficient; SE = standard error; t-value = *t*-test statistic; *p*-value = statistical significance level. *p*-values less than 0.05 are considered statistically significant and are indicated in bold in the table. Binary categorical variables are coded as 1 = yes/0 = no; EWLR = expected weight loss rate; GAD-7 = Generalized Anxiety Disorder 7-item scale, classified as mild, moderate, or severe; PHQ-9 = Patient Health Questionnaire 9-item scale, classified as mild, moderate, or severe.

## Data Availability

The datasets used and/or analyzed during the current study are available from the corresponding authors on reasonable request.

## Abbreviations

The following abbreviations are used in this manuscript:

BMI	body mass index
EWLR	expectations regarding weight loss
CIA	Clinical Impairment Assessment
PHQ-9	Patient Health Questionnaire-9
GAD-7	Generalized Anxiety Disorder Questionnaire-7
IQR	interquartile range
OLS	ordinary least squares
BIC	Bayesian Information Criterion
LRT	likelihood ratio test

## References

[1] Sarwer D.B., Heinberg L.J. (2020). A Review of the Psychosocial Aspects of Clinically Severe Obesity and Bariatric Surgery. Am. Psychol..

[2] Hales C.M., Fryar C.D., Carroll M.D., Freedman D.S., Aoki Y., Ogden C.L. (2018). Differences in Obesity Prevalence by Demographic Characteristics and Urbanization Level Among Adults in the United States, 2013–2016. JAMA.

[3] Deledda A., Pintus S., Loviselli A., Fosci M., Fantola G., Velluzzi F. (2021). Nutritional Management in Bariatric Surgery Patients. Int. J. Environ. Res. Public Health.

[4] van Hout G.C.M., van Oudheusden I., van Heck G.L. (2004). Psychological Profile of the Morbidly Obese. Obes. Surg..

[5] Courcoulas A.P., King W.C., Belle S.H., Berk P., Flum D.R., Garcia L., Gourash W., Horlick M., Mitchell J.E., Pomp A. (2018). Seven-Year Weight Trajectories and Health Outcomes in the Longitudinal Assessment of Bariatric Surgery (LABS) Study. JAMA Surg..

[6] Martinelli V., Chiappedi M. (2022). Bariatric Surgery: Psychosocial Aspects and Quality of Life. Int. J. Environ. Res. Public Health.

[7] Sarwer D.B., Allison K.C., Bailer B., Faulconbridge L.F., Wadden T.A. (2013). Bariatric Surgery. Presurgical Psychological Screening: Understanding Patients, Improving Outcomes.

[8] Maraldo T.M., Fewell L., Vander Wal J.S. (2021). Factor Structure and Psychometric Properties of the Clinical Impairment Assessment 3.0 (CIA) in a Clinical Eating Disorder Sample. Eat. Behav..

[9] Sarwer D.B., Polonsky H.M. (2016). The Psychosocial Burden of Obesity. Endocrinol. Metab. Clin. N. Am..

[10] Heinberg L.J., Ashton K., Windover A. (2010). Moving beyond Dichotomous Psychological Evaluation: The Cleveland Clinic Behavioral Rating System for Weight Loss Surgery. Surg. Obes. Relat. Dis..

[11] Marek R.J., Heinberg L.J., Lavery M., Merrell Rish J., Ashton K. (2016). A Review of Psychological Assessment Instruments for Use in Bariatric Surgery Evaluations. Psychol. Assess..

[12] De Luca M., Angrisani L., Himpens J., Busetto L., Scopinaro N., Weiner R., Sartori A., Stier C., Lakdawala M., Bhasker A.G. (2016). Indications for Surgery for Obesity and Weight-Related Diseases: Position Statements from the International Federation for the Surgery of Obesity and Metabolic Disorders (IFSO). Obes. Surg..

[13] (2013). Presurgical Psychological Screening: Understanding Patients, Improving Outcomes.

[14] (2018). Handbook of Obesity Treatment.

[15] Sogg S., Lauretti J., West-Smith L. (2016). Recommendations for the Presurgical Psychosocial Evaluation of Bariatric Surgery Patients. Surg. Obes. Relat. Dis..

[16] Mechanick J.I., Youdim A., Jones D.B., Garvey W.T., Hurley D.L., McMahon M.M., Heinberg L.J., Kushner R., Adams T.D., Shikora S. (2013). Clinical Practice Guidelines for the Perioperative Nutritional, Metabolic, and Nonsurgical Support of the Bariatric Surgery Patient—2013 Update: Cosponsored by American Association of Clinical Endocrinologists, the Obesity Society, and American Society for Metabolic & Bariatric Surgery. Endocr. Pract..

[17] Puhl R.M., Heuer C.A. (2009). The Stigma of Obesity: A Review and Update. Obesity.

[18] Puhl R.M., Heuer C.A. (2010). Obesity Stigma: Important Considerations for Public Health. Am. J. Public Health.

[19] Calugi S., Sartirana M., Milanese C., El Ghoch M., Riolfi F., Dalle Grave R. (2018). The Clinical Impairment Assessment Questionnaire: Validation in Italian Patients with Eating Disorders. Eat. Weight. Disord..

[20] Weiss F., Barbuti M., Carignani G., Calderone A., Santini F., Maremmani I., Perugi G. (2020). Psychiatric Aspects of Obesity: A Narrative Review of Pathophysiology and Psychopathology. J. Clin. Med..

[21] Bohn K., Doll H.A., Cooper Z., O’Connor M., Palmer R.L., Fairburn C.G. (2008). The Measurement of Impairment Due to Eating Disorder Psychopathology. Behav. Res. Ther..

[22] Jenkins P.E. (2013). Psychometric Validation of the Clinical Impairment Assessment in a UK Eating Disorder Service. Eat. Behav..

[23] Dahlgren C.L., Stedal K., Rø Ø. (2017). Eating Disorder Examination Questionnaire (EDE-Q) and Clinical Impairment Assessment (CIA): Clinical Norms and Functional Impairment in Male and Female Adults with Eating Disorders. Nord. J. Psychiatry.

[24] Spitzer R.L., Kroenke K., Williams J.B. (1999). Validation and Utility of a Self-Report Version of PRIME-MD: The PHQ Primary Care Study. Primary Care Evaluation of Mental Disorders. Patient Health Questionnaire. JAMA.

[25] Kroenke K., Spitzer R.L., Williams J.B. (2001). The PHQ-9: Validity of a Brief Depression Severity Measure. J. Gen. Intern. Med..

[26] Löwe B., Decker O., Müller S., Brähler E., Schellberg D., Herzog W., Herzberg P.Y. (2008). Validation and Standardization of the Generalized Anxiety Disorder Screener (GAD-7) in the General Population. Med. Care.

[27] Rosenbaum D.L., White K.S. (2016). Understanding the Complexity of Biopsychosocial Factors in the Public Health Epidemic of Overweight and Obesity. Health Psychol. Open.

[28] Kyrou I., Tsigos C. (2007). Stress Mechanisms and Metabolic Complications. Horm. Metab. Res..

[29] Pyykkö J.E., Aydin Ö., Gerdes V.E.A., Acherman Y.I.Z., Groen A.K., van de Laar A.W., Nieuwdorp M., Sanderman R., Hagedoorn M. (2022). Psychological Functioning and Well-Being before and after Bariatric Surgery; What Is the Benefit of Being Self-Compassionate?. Br. J. Health Psychol..

[30] Puhl R., Suh Y. (2015). Health Consequences of Weight Stigma: Implications for Obesity Prevention and Treatment. Curr. Obes. Rep..

[31] Gonçalves I.d.S.A., Filgueiras M.D.S., Moreira T.R., Thomé M.S., Paiva G.L.D., Almeida G.P.d., Cotta R.M.M., Campos T.d.N., Freitas D.M.d.O., Novaes J.F.d. (2024). Interrelation of Stress, Eating Behavior, and Body Adiposity in Women with Obesity: Do Emotions Matter?. Nutrients.

[32] Causes of Emotional Eating and Matched Treatment of Obesity-PubMed. https://pubmed.ncbi.nlm.nih.gov/29696418/.

[33] Simon G.E., Von Korff M., Saunders K., Miglioretti D.L., Crane P.K., van Belle G., Kessler R.C. (2006). Association between Obesity and Psychiatric Disorders in the US Adult Population. Arch. Gen. Psychiatry.

[34] Balantekin K.N., Grammer A.C., Fitzsimmons-Craft E.E., Eichen D.E., Graham A.K., Monterubio G.E., Firebaugh M.-L., Karam A.M., Sadeh-Sharvit S., Goel N.J. (2021). Overweight and Obesity Are Associated with Increased Eating Disorder Correlates and General Psychopathology in University Women with Eating Disorders. Eat. Behav..

[35] van Hout G.C.M., Boekestein P., Fortuin F.A.M., Pelle A.J.M., van Heck G.L. (2006). Psychosocial Functioning Following Bariatric Surgery. Obes. Surg..

[36] Wingo B.C., Desmond R.A., Brantley P., Appel L., Svetkey L., Stevens V.J., Ard J.D. (2013). Self-Efficacy as a Predictor of Weight Change and Behavior Change in the PREMIER Trial. J. Nutr. Educ. Behav..

[37] Cohen A.K., Rai M., Rehkopf D.H., Abrams B. (2013). Educational Attainment and Obesity: A Systematic Review. Obes. Rev..

[38] Patsalos O., Keeler J., Schmidt U., Penninx B.W.J.H., Young A.H., Himmerich H. (2021). Diet, Obesity, and Depression: A Systematic Review. J. Pers. Med..

